# Activated Metals to Generate Heat for Biomedical Applications

**DOI:** 10.1021/acsmaterialslett.3c00581

**Published:** 2023-08-16

**Authors:** Eva Remlova, Vivian Rachel Feig, Ziliang Kang, Ashka Patel, Ian Ballinger, Anna Ginzburg, Johannes Kuosmanen, Niora Fabian, Keiko Ishida, Joshua Jenkins, Alison Hayward, Giovanni Traverso

**Affiliations:** † Division of Gastroenterology, Department of Medicine, Brigham and Women’s Hospital, Harvard Medical School, Boston, Massachusetts 02115, United States; ‡ The David H. Koch Institute for Integrative Cancer Research, Massachusetts Institute of Technology, Cambridge, Massachusetts 02139, United States; § Department of Health Sciences and Technology, Eidgenössische Technische Hochschule Zürich, Universitätstrasse 2, 8092 Zürich, Switzerland; ∥ Department of Mechanical Engineering, Massachusetts Institute of Technology, Cambridge, Massachusetts 02139, United States; ⊥ Department of Bioengineering, Northeastern University, Boston, Massachusetts 02115, United States; # Department of Cell/Cellular and Molecular Biology, Northeastern University, Boston, Massachusetts 02115, United States; ○ Division of Comparative Medicine, Massachusetts Institute of Technology, Cambridge, Massachusetts 02139, United States

## Abstract

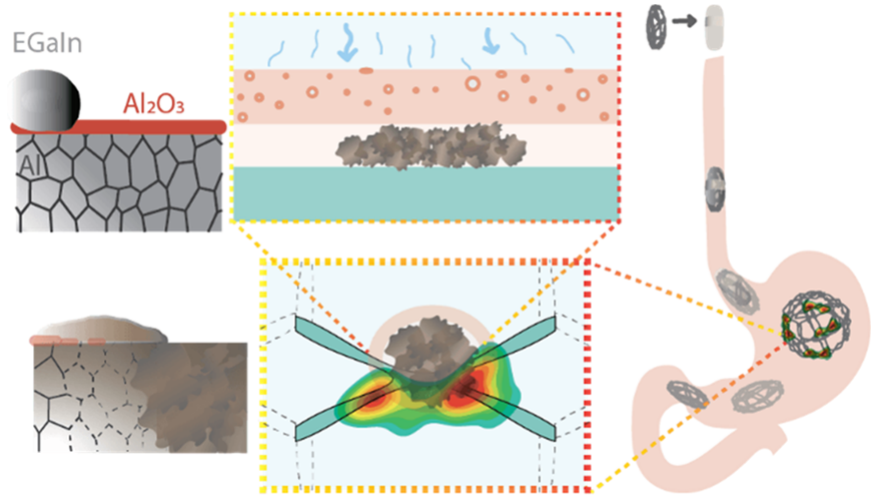

Delivering heat *in vivo* could enhance a wide range of biomedical therapeutic and diagnostic technologies, including long-term drug delivery devices and cancer treatments. To date, providing thermal energy is highly power-intensive, rendering it oftentimes inaccessible outside of clinical settings. We developed an *in vivo* heating method based on the exothermic reaction between liquid-metal-activated aluminum and water. After establishing a method for consistent activation, we characterized the heat generation capabilities with thermal imaging and heat flux measurements. We then demonstrated one application of this reaction: to thermally actuate a gastric resident device made from a shape-memory alloy called Nitinol. Finally, we highlight the advantages and future directions for leveraging this novel *in situ* heat generation method beyond the showcased example.

Low power methods of delivering heat *in vivo* that do not require an external power source or an onboard galvanic cell would benefit several treatment and diagnostic modalities that rely on thermal energy either directly or indirectly. As a direct form of therapy, heat has been explored for cancer therapy, thermal therapy for chronic wounds, arthritis, and more.^1−4^ Indirectly, heat can be used to trigger drug release from thermoresponsive matrices^4−8^ and to actuate thermally responsive robotic actuators to perform functions like biopsy and single-cell manipulation.^9−14^

Conventional heating methods can broadly be grouped into two categories, and both have challenges that limit their potential to be employed in out-of-hospital settings (Supplementary Table 1). The first group harnesses external fields—including magnetic, ultrasound, and electric fields—to interact with thermally responsive materials inside the body.^15−18^ However, significant power is typically required for field generation, especially considering attenuation through tissue, bone, and air pockets, and these are therefore challenging to incorporate into portable systems outside of the clinic.^1^ The second group leverages exothermic chemical reactions including acid–base neutralization, alkali metal-water reactions, and common salt-water reactions (Table 1) and has been demonstrated for tumor ablation^19−21^ and thermoembolization.^22^ Yet, these reactions tend to suffer from poor spatial specificity; require large volumes or high concentrations to generate significant heat; or form byproducts that can potentially damage adjacent tissue.^19^

**Table 1:**
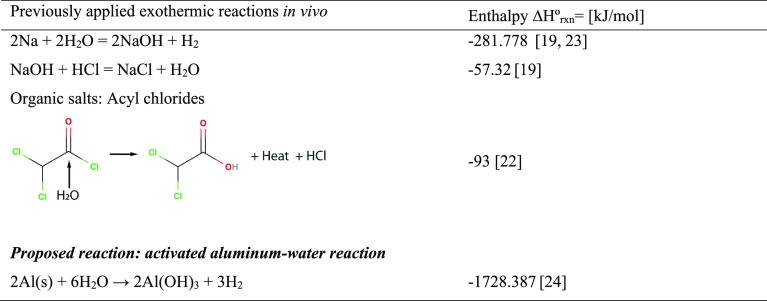
Examples of Exothermic Reactions *in Vivo* and Their Respective Enthalpies

We propose a novel biomedical heat generation method based on activated aluminum (Al), which reacts with water in a highly exothermic reaction that theoretically releases substantially more heat (1728.387 kJ/mol) at body temperature than any of the previously explored chemical methods for generating heat *in vivo* (Table 1), enabling it to be introduced in small amounts to generate heat with low or no power. Moreover, Al can be readily patterned using conventional device fabrication techniques and is therefore potentially more easily incorporated into existing workflows for manufacturing complex bioelectronics or robotics systems. Al metal is also fairly biocompatible^25^ and consumed regularly in small amounts through dietary and pharmaceutical sources.^26,27^ The toxicity of various forms of Al is largely determined by their physical characteristics and their solubilities in water. The toxicity of soluble Al forms is mainly dependent on the amount of Al 3+ ions delivered to target tissues.^28^ Al 3+ is a favored cation for many ligands due to its significant biological reactivity, and there is no universal threshold as its toxicity depends on its interactions with ligands in a specific environment, varying in different biological compartments with their own signature ligands for binding Al 3+.^27^ Conversely, the toxicity of insoluble Al oxides is predominantly determined by their behavior as particulates.^28^ The US Agency for Toxic Substances and Disease Registry (2008) derives a minimal risk level of 1 mg Al/kg/day for intermediate-duration oral exposure (15–364 days).^29^

Under normal circumstances, Al does not react with water because it is passivated by a thin self-healing Al oxide layer.^30^ While strongly acidic or basic environments can destabilize and remove the oxide,^31^ resulting in an active Al state that is reactive with water, these are impractical and potentially unsafe to introduce within the body. Here, we used liquid metal eutectic gallium indium (EGaIn) to activate Al in a biocompatible manner so that it can react exothermically with water under physiological conditions. Various studies have established the biosafety of gallium alloys at different doses and in different dosage forms.^34−37^ Accordingly, liquid EGaIn^38^ has been researched for many biointerfacing applications, ranging from printable^39^ and stretchable electronics^40^ to nanomedicine^37,41,42^ and drug delivery.^43−46^

Gallium alloys like EGaIn both depassivate Al and prevent passivation by penetrating its grain boundaries. As soon as a critical surface concentration of Ga in the liquid state on Al is achieved, wetting of Al facilitates the formation of a Ga–Al amalgam. Its surface diffusion is responsible for detachment of the Al oxide film and a shift of the electrochemical potential of Al to more negative values, resulting in a uniform attack morphology. The exothermic Al ion hydrolysis promotes this process, while the loss of Ga at the active interface hinders it.^31^ Depassivated Al at body temperature reacts exothermically with water via the following reactions:^32,33,24^
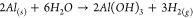
1
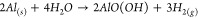
2

The reaction between Al and water at room temperature has a calculated standard molar reaction enthalpy of 1728.387 kJ/mol.^24^ That makes activated Al considerably more exothermic than any of the previously applied *in vivo* exothermic reactions discussed in Table 1.

In this article, we identify the steps necessary to successfully leverage heat from the EGaIn-activated Al-water reaction and demonstrate one potential application in a thermally responsive gastric device (Figure 1).

**Figure 1:**
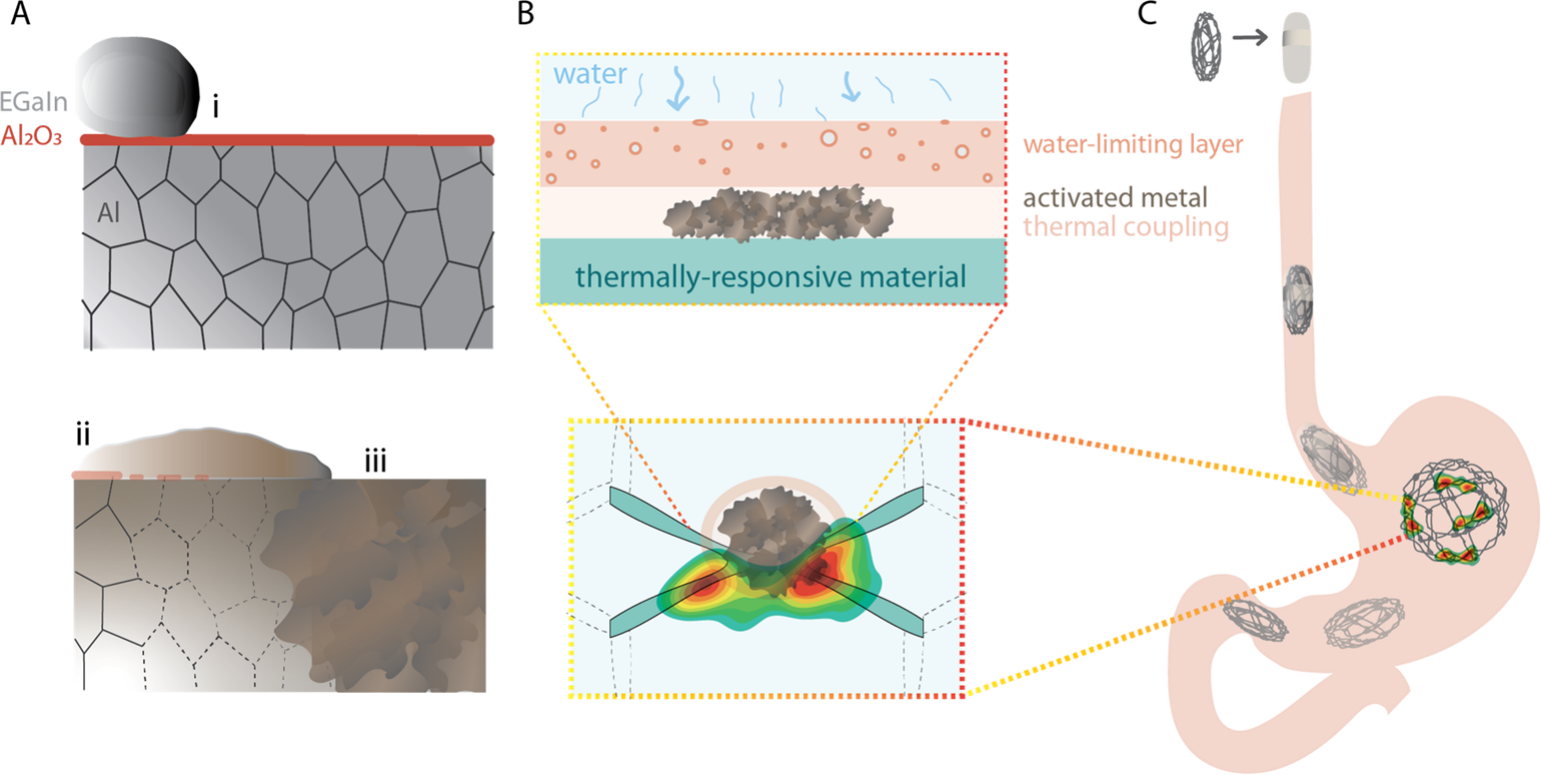
Activated aluminum to generate heat: (A) Al-grain boundaries penetration by EGaIn: (i) amalgamation is prevented by Al oxide, (ii) Al-surface wetting leads to EGaIn penetrating the grain boundaries, (iii) amalgamation and activated metal formation, (B) cross-sectional view of the building components to leverage the heat generation, and (C) proposed actuation of a gastrointestinal device.

**1. Requirements to effectively harness heat from activated aluminum**. To assess the heat generation capabilities of activated Al, we first contacted a 2 cm^2^ Al film with oxidized EGaIn, which promotes wetting of Al and formation of a Ga–Al amalgam.^47^ Successful wetting was indicated by a change in color of EGaIn, signaling the formation of the Ga–Al amalgam (Supplementary Figure 1).^48−51^ After letting the mixture equilibrate for 2 h, we exposed the samples to roughly 2 mL of water and recorded a maximum surface temperature of 120.7 °C via an IR camera over the course of 1.5 min (Figure 2A). For potential tissue-interfacing applications, we also recorded the surface temperature of the reaction encapsulated in a semipermeable membrane (Supplementary Discussion 1, Supplementary Figure 2) as well as the impact of pH and buffering on the activated Al-water reaction (Supplementary Discussion 2, Supplementary Figures 3 and 4). Next, a heat flux sensor was used to quantify the flow of the thermal energy from the reaction. To set up the heat flux measurements (Supplementary Figure 5), an Al plate was used as a heat sink, and Kapton tape and Styrofoam provided insulation. We found that using thermal grease and modeling clay was important to establish sufficient thermal contact between Al and the sensor. Afterward, water was introduced via a syringe through the insulation. With this setup, we recorded heat flux data to study the following variables: the exposure time of Al to EGaIn, at 20% relative humidity (RH); different weight ratios of EGaIn to Al; volume of added water; and rate of addition.

**Figure 2:**
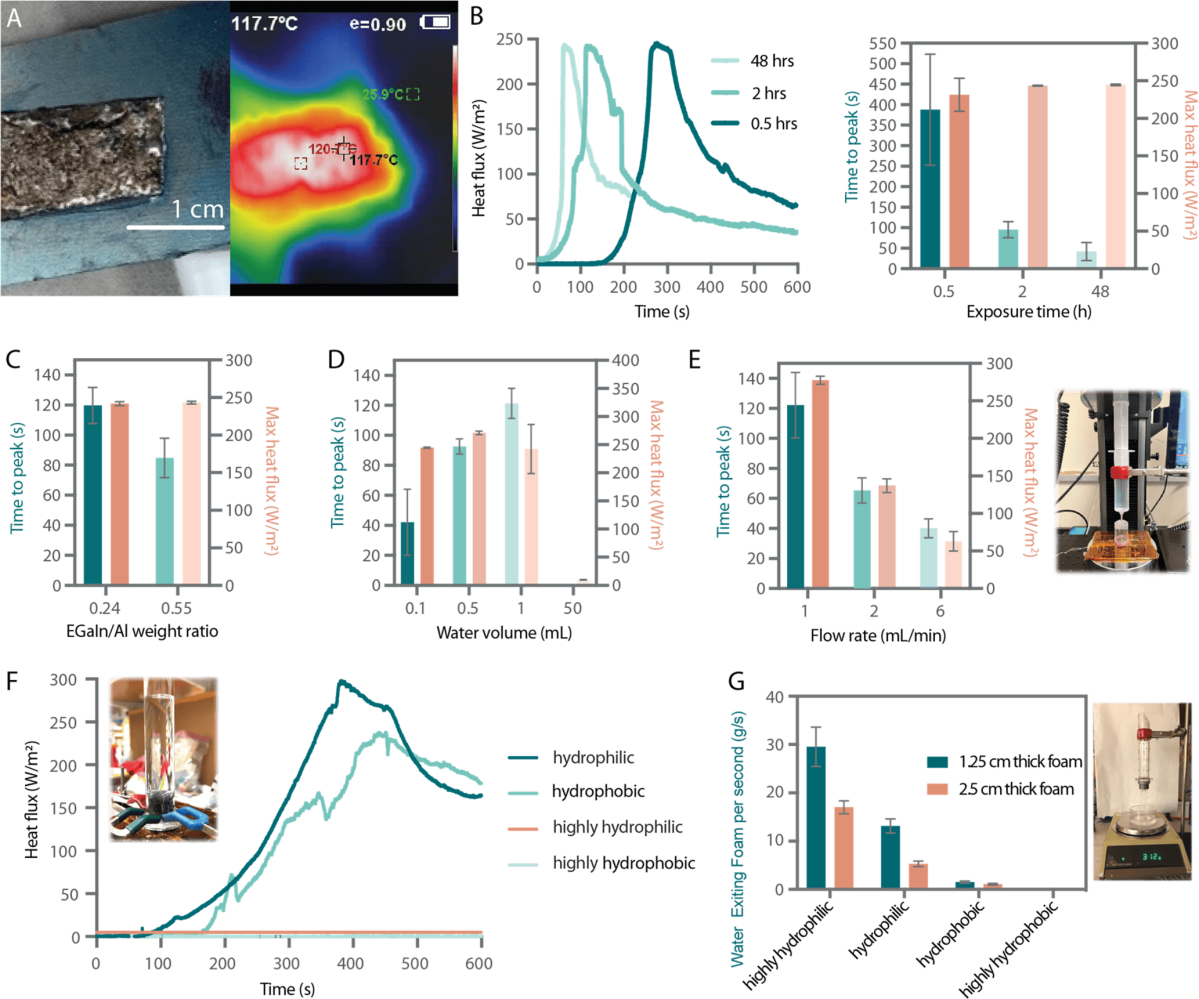
Sample setup and results: (A) thermal camera imaging measurement of activated aluminum when exposed to water, (B) the effect of varying exposure time to RH on the heat flux maximum and time to reach the maximum, (C) the impact of different weight ratios and varying water volume, (D, E) heat flux setup for three different flow rates of water, (F) average mass flow of water through various foams of two different thicknesses and the respective setup, and (G) experimental setup for mimicking a fully submerged environment with controlled, slowed down water penetration using foam materials and the respective heat flux data.

The maximum recorded heat flux was consistent for different exposure times of Al to EGaIn under ambient conditions before the addition of water (Figure 2B). However, samples that were left treated with EGaIn for the longest time also took the shortest time to reach that maximum, which could be explained by a higher degree of grain boundary saturation. Varying weight ratios of EGaIn to Al again did not change the maximum heat flux achieved, while larger ratios took less time to reach that maximum (Figure 2C).

We also observed that both the amount and the rate of water exposure are important for sufficient heat generation. When activated Al was flooded with water, hardly any heat was generated (Figure 2D), which could be attributed to competition between heat generation and heat dissipation to the water around the sensor, which acts as a heat sink. Additionally, the maximum heat flux decreased with an increasing water flow rate (Figure 2E). The time to reach the maximum value also decreased slightly as the water flow rate increased, reflecting the fact that water acts both as a reactant in the exothermic Al-water reaction and as a heat sink.

In several biomedical applications, devices are likely to be submerged in an aqueous environment. To maintain a constant source of water while slowing heat dissipation, we hypothesized that porous materials such as foams would reduce the rate of water introduced to the reactant. To confirm this, we set up experiments with foams of different degrees of hydrophobicity and designed a custom setup (Figure 2F) for these measurements by attaching a flange tube to the sensor and sealing the connection with an O-ring, with the entire setup held in place by clamping the flange to the heatsink. While the setups with the hydrophobic and fully permeable foams generated negligible heat, the hydrophilic foams were semipermeable to water and thus allowed us to recapitulate the same amount of heat that could be generated in air (Figure 2F). We then designed a custom setup to further measure the water flow rates through the different foams (Supplementary Figure 6) and observed that the moderately hydrophilic foam associated with the highest heat flux values also corresponded to intermediate water flow rates. Measuring the water flow through foams of various hydrophobicities using a custom setup (Supplementary Figure 6) revealed that the medium hydrophilic foam is also linked to the highest heat flux values recorded (Figure 2G). Therefore, limiting the rate of water penetration above a minimum threshold volume is key to maximizing heating performance.

Based on the above experiments, we established three main requirements to be able to effectively leverage heat generation from the Al-water reaction (Figure 1): First, since Al must be in an activated form to generate heat, complete saturation of Al by EGaIn should be promoted by increasing either the exposure time or the EGaIn quantity. Sufficient thermal coupling is also critical to effectively couple the heat generated to a thermoresponsive material, as evidenced by the importance of thermal glue in obtaining high-quality heat flux measurements. Finally, limiting the rate of water entering the system is critical and can be achieved with no external power source by incorporating a semipermeable porous membrane.

**2. Actuating a thermally responsive gastric device**. To demonstrate how these design principles can be applied to biomedical robotic devices, we designed an orally administrable, shape-changing gastric resident device inspired by the Hoberman sphere that harnesses activated Al and the shape memory metal nickel titanium alloy^52^ (Nitinol) to collapse for on-demand end-of-life elimination (Figure 3A). Several gastroretentive robotic systems have been developed by our group for long-term drug delivery and sensing; to further their translational potential, there is a demand for mechanisms to eliminate these devices from the stomach on demand without requiring invasive interventions for device retrieval.

**Figure 3:**
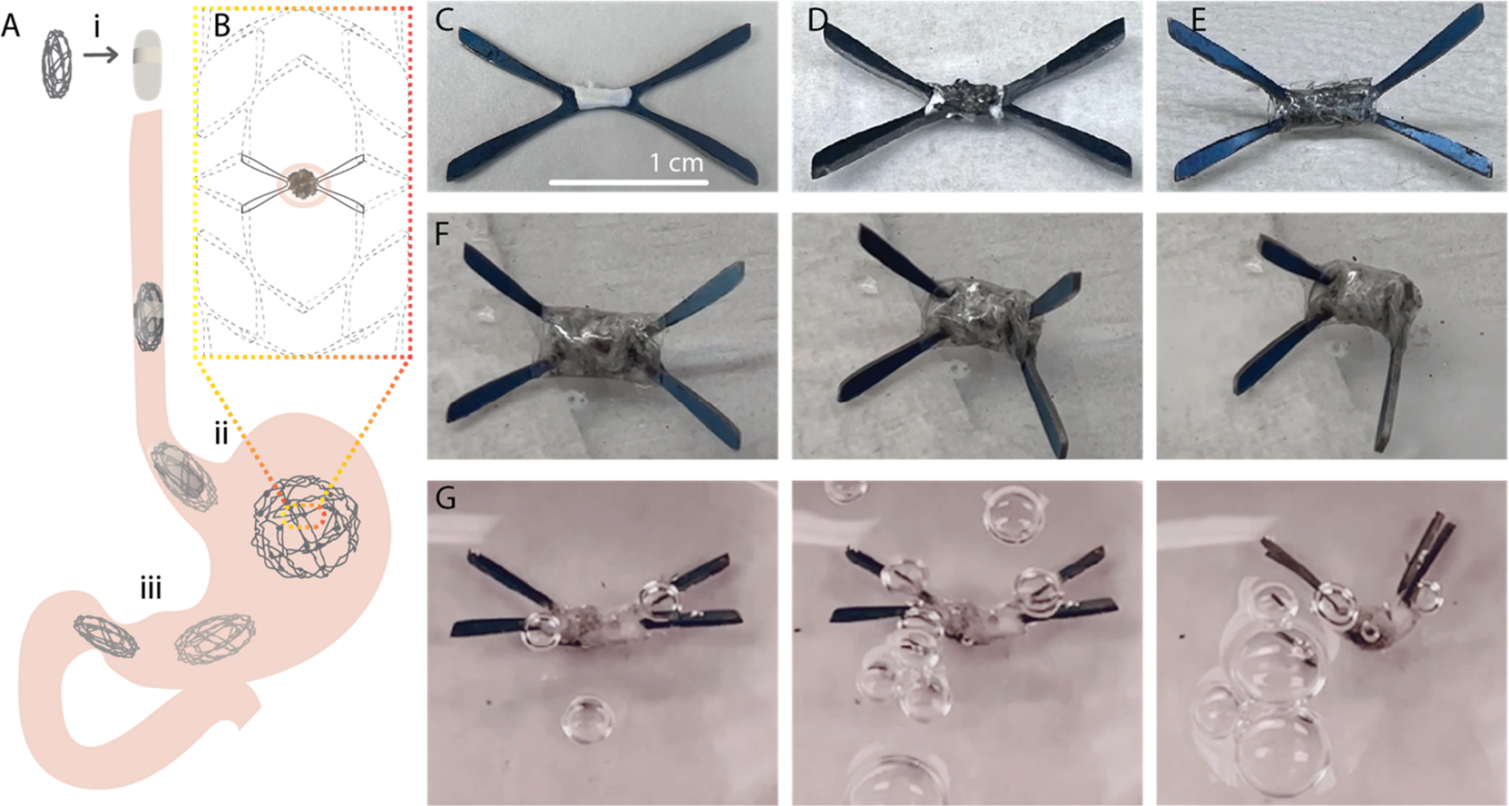
Gastrointestinal (GI) device requirements and hinge functionality: (A) fundamental design requirements of an ingestible GI device, demonstrated on a collapsible device prototype for noninvasive elimination: (i) fit inside a swallowable capsule, (ii) successful deployment, (iii) noninvasive elimination, (B) laser-cut hinge close-up within the device design, (C) application of thermal grease to provide thermal coupling, (D) adding activated Al powder, (E) dialysis tubing wrapped around the center of the hinge, (F) actuation in air, and (G) actuation when submerged in room temperature water.

For oral administration, devices should fit within a swallowable 000 capsule (1.37 mL, 26.14 mm × 9.91 mm).^52^ After administration, the device changes shape to exceed a diameter of 2 cm, which is required for retention via luminal confinement.^52−56^ Our design comprised numerous thermally responsive Nitinol hinges that would enable the system to collapse and be eliminated from the stomach upon actuation. We focused on optimizing a single hinge component, which serves as the building block to engineer more complex device designs in the future (Figure 3B, Supplementary Discussion 3 and Supplementary Video 1).

First, as a proof of concept that the EGaIn-activated Al-water reaction could generate sufficient heat for Nitinol actuation, we set a 1 mm diameter Nitinol wire into the shape of a torsion spring using a custom fixture (Supplementary Figure 7C) for annealing and subsequent quenching. The arms of the spring were pulled open and then wrapped with activated Al wire that had previously been smeared with EGaIn; the preprogrammed shape of the Nitinol spring was recovered upon adding a few drops of water (Supplementary Figure 7A-B).

To inform the design of the Nitinol hinge, topology optimization was performed to minimize the required heating area needed for successful actuation, taking advantage of the fact that Nitinol is thermally conductive. For the simulations, a heat flux value of 200 W/m^2^ was used based on the typical maxima observed from our heat flux measurements. In solving the topology optimization problem, we sought to find the optimal geometrical configuration of a Nitinol hinge by maximizing its displacement under this heat flux, subject to several constraints described in Supplementary Discussion 3. To save computational power, we treated this as a symmetric design problem (Supplementary Figures 8A and 8B, Supplementary Video 2). Mirroring the resulting Nitinol configuration yielded the optimized 4-arm hinge design (Supplementary Figure 8C), which achieves flexure joint functionality when heated solely from the center.

After laser cutting Nitinol into the optimized hinge shape (Supplementary Figure 9A), we set it into a collapsed state by annealing it while it was clamped and folded (Supplementary Figure 9B). To integrate the hinge with our heat generation mechanism, we applied the basic design requirements elucidated from our heat flux measurements: First, complete Al activation was ensured by saturating it with excess EGaIn for 2 days, as evidenced by the transformation of Al into a loose powder. The activated Al was thermally coupled to the center of the Nitinol hinge (Figure 3D) using thermal grease (Figure 3C), and dialysis tubing was wrapped around the center to limit the rate of water penetration and potentially also provide thermal insulation (Figure 3E). Here, dialysis tubing was also sufficient to contain the reaction products (Figure 3G). For a 10,000 molecular weight-cutoff, the pore size is on average roughly 30 Å,^57^ compared to typical metal grain sizes that are 1 μm or larger.^58^ The hinge was successfully actuated by dropping water on it in air (Figure 3F) as well as upon full immersion in room temperature water (Figure 3G). Actuation upon water contact in air and underwater took 15 and 45 s, respectively. Notably, the hinge was unable to actuate in water when dialysis tubing was not used, emphasizing the importance of including a water-limiting layer in the design.

Building on these results, we next assembled an orally administrable prototype using superelastic Nitinol to build deployable 5 cm-long arms that we attached to the central hinge structure (Figure 4A). We chose superelastic Nitinol due to its robustness and ability to recover its shape after being folded for a long period of time inside the capsule. In the deployed state, all dimensions of the prototype exceeded the 2 cm diameter of the pyloric sphincter, as needed for retention in the stomach (Figure 4A).^53^

**Figure 4:**
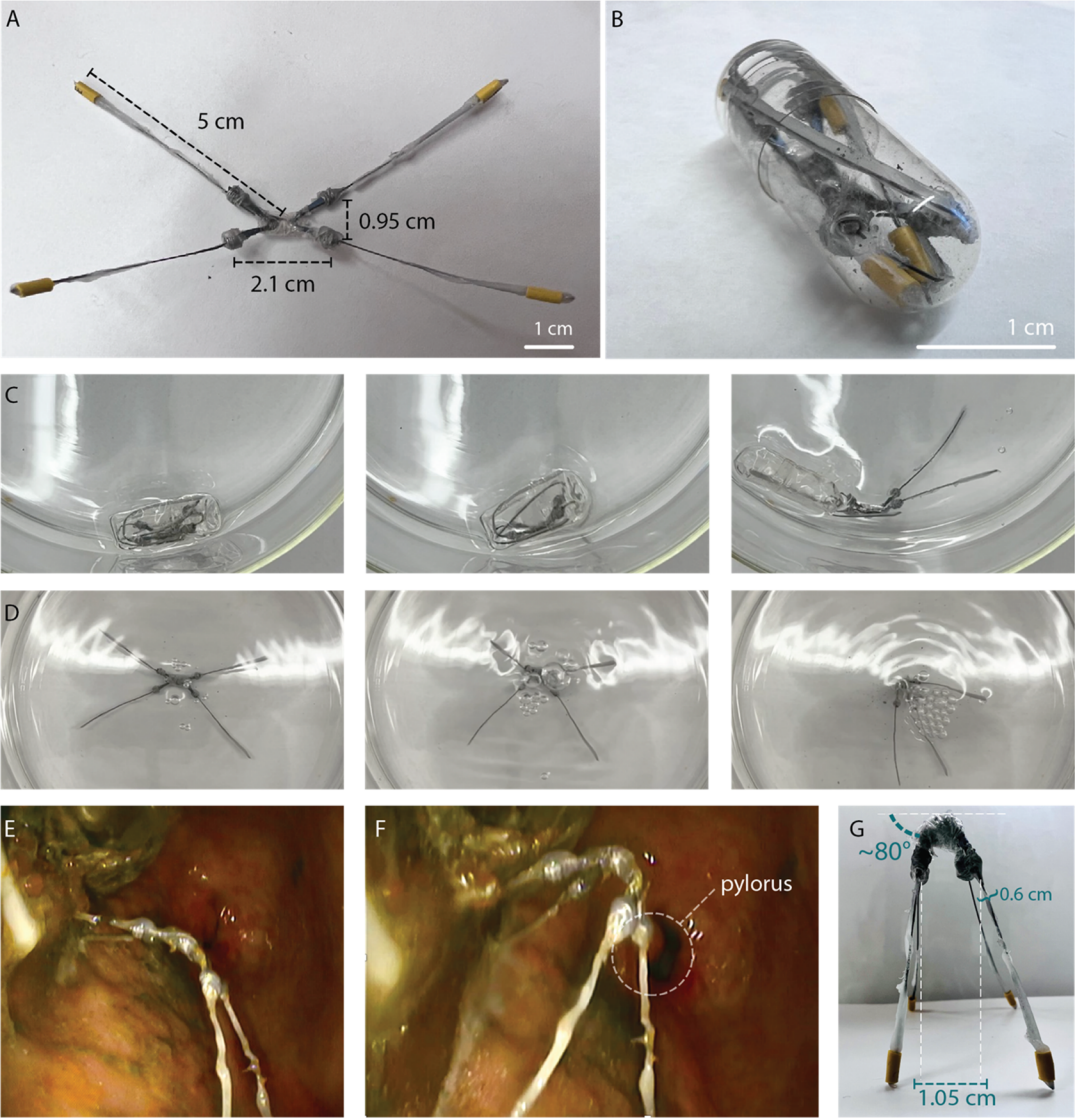
Device demo *in vitro* and *in vivo*: Prototype assembly: (A) deployable superelastic Nitinol arms coated with PCL, application of thermal grease, activated Al powder adhered to the center of the hinge, final assembly with dialysis tubing and heat shrink tubing around the ends of the arms to promote visibility for *in vivo* experiments, (B) fitting the prototype inside a 000 capsule by folding *ex vivo* and *in vivo* validations, (C) deployment from capsule, (D) actuation in room temperature water, (E) esophageal endoscopy-assisted capsule insertion *in vivo* and prototype deployment from a 000 capsule, (F) actuation, and (G) folding angles after actuation.

Next, we placed the activated Al powder in the middle of the Nitinol hinge component (Figure 4A) and folded the prototype to fit inside a 000 capsule (Figure 4B). Deployment of the prototype from the capsule (Figure 4C) was confirmed *ex vivo* upon submergence in room temperature water, followed by successful actuation and refolding based on the reaction of water with the activated Al (Figure 4D). The prototype was also validated *in vivo* in the stomach of an anesthetized pig. The capsule containing the device was able to be introduced into the stomach via an overtube (Figure 4E), and the prototype deployed within 10 s of the capsule dissolving within the gastric fluid (Figure 4E). In the deployed state, the activated Al was able to react with the moisture in the gastric cavity, and actuation and refolding occurred within approximately 25 s (Figure 4F).

To confirm its capacity for safe passage after actuation, we measured the hinge width and folding angle for both the deployed and folded states of the two prototypes to further assess their performance. For the deployed state (Figure 4A), the dimensions of the hinge were 2.1 cm, compared to 1.35 and 1.05 cm when folded (Figure 4G). Assuming symmetric folding, the folding angles for each arm of the two prototypes were calculated to be 65° and 80°, respectively. The folding angles are dictated by the hinge design, the processing of the Nitinol, and the grade of Nitinol and can be increased to 100° or beyond.^59,60^

**3. Advantages compared to alternative heating schemes**. Illustrating the energy efficiency of our chemical heating method, we compared the heat generation capabilities of activated Al to the total energy stored in batteries that are conventionally used for ingestible devices. Referencing specifications (Supplementary Table 2) indicated for a silver oxide coin cell battery that fits inside an ingestible 000 capsule, we calculated that a total of about 2.4 kJ of heat can be generated if the entire capsule were filled with 15 batteries. In our device, we used about 15 mg of Al, which can provide 480 J of energy when reacted with water—about three times as much energy as is stored in a single battery. Similarly, we would only need 75 mg of Al for the 2.4 kJ energy equivalent of 15 batteries. These calculations highlight the promising potential for miniaturization by using this highly exothermic heating method.

Future work will entail investigating methods that increase temporal control over the heat generation method. Device elements that passively introduce water after a predefined time period, including either degradable membranes or osmotic pumps, could be incorporated. Additional complexity can be introduced by leveraging advances in stimuli-responsive materials, such as self-immolating polymers^61^ and smart gating membranes.^62^ Next-generation device designs could also enable other actuation mechanisms, such as electrical and magnetic triggering, to be transduced into heating via the Al-water reaction. This would enable low-power actuation schemes in which our chemical heating method amplifies the responses enabled by a variety of external stimuli. For example, rather than using Joule heating alone to stimulate a thermally responsive actuator, considerably less power could be used to dissolve a pinhole in a membrane that lets in sufficient water to drive the activated-Al-water reaction.

To showcase the power amplification potential of our heating mechanism, we calculated how much power would be required to actuate the Nitinol component of our prototype using an electrical power supply versus a low-power alternative consisting of activated Al. To determine power requirements, we used the following equation: P = E·t, where P stands for power, E is energy, and t is time in seconds. Nitinol has a latent heat of transformation of approximately 20 J/g and a specific heat of 0.01 J/g*C.^63^ Due to the competition between heating and cooling via heat dissipation to surroundings, we assumed that Nitinol should be actuated within 2 s.

We determined that 2.55 W of power is required to generate sufficient heat for Nitinol actuation using an electrical current (Supplementary Table 3). By comparison, heating with activated Al could be triggered on demand using electrical power to dissolve a thin membrane instead. For example, gold, which is otherwise inert in an acidic environment, can be electrochemically corroded by shifting the electrochemical potential. It has been previously shown that using a 300 nm thick gold membrane to seal a model drug reservoir system, 0.8 mW of power was required to corrode the membrane electrically for on-demand drug release.^9^ Thus, combining an electrochemically corroded membrane with EGaIn-activated aluminum could enable power amplification of 3,190×.

We developed a novel low-power heat generation scheme for broad biomedical applications using the reaction between EGaIn-activated Al and water. Because of its high volumetric energy density, our proposed solution is highly efficient and readily miniaturized. As a proof of concept to illustrate potential future applications, we used an activated Al system to thermally actuate a gastric resident device. In the future, this heating mechanism can be incorporated into more complex systems, including devices that use actively triggerable elements to initiate heating on-demand. Because of its high power efficiency, high spatial resolution, and compatibility with device designs that improve controllability and complexity, we anticipate that heating with activated metals can open the door to new therapeutic uses of heat.

## Methods

### Materials Selection

Throughout all experiments, EGaIn (75.5% Ga/24.5% In, PubChem SID 2487 2973) was purchased from Sigma-Aldrich, and 99.6% pure Al sheet stock (1060 Al, 0.2 mm thickness) was obtained from Yodaoke. Heat flux measurements were set up using Kapton tape, Styrofoam, and thermal silicone grease (TG-S606P) purchased from Digi-Key and polymer clay purchased from Craft Smart. For measurements incorporating foam, polyethylene cross-linked closed-cell sheet (3/4 in., Item #5GCK2), polyurethane open cell (1/8 in., Item #5GCW6), and polyurethane open cell (1/2 in., Item #5GCG3) foams were obtained from Grainger, Inc., and melamine foam (0.8 in., Trendbox) was purchased from Amazon. For prototyping, shape-memory Nitinol wire (1 mm, transition temperature 80°) and sheets (various thicknesses: 0.15, 0.25, 0.5, 0.75, and 1 mm, transition temperature 45 °C), as well as superelastic Nitinol wire (0.25 mm), were purchased from Kellogg’s Research Laboratories. Al wire (4 oz., 20 AWG, #470123-844) was obtained from VWR, and dialysis tubing (10,000 MWCO) was obtained from Fisher Scientific. Polycaprolactone (PCL) (50000 MW, CAS #24980-41-4) pellets were purchased from Polysciences, Inc. Lastly, heat shrink tubing (125 °C, ø 2.0, Eventronic), superglue (Loctite 4851 instant adhesive), and gelatin capsules (size 000, PureCaps USA) were obtained from Amazon.

### Consistent Aluminum Activation

Al was activated by stirring EGaIn with a pipet tip by hand on the surface of the Al until wetting occurred. Qualitative changes, including surface discoloration upon activation, were visually analyzed and accordingly documented.

### Heat Characterization

The heat generation capabilities of activated Al were evaluated by using a Perfect-Prime Thermal Imaging Camera IR001851 to capture the maximal temperature generated by the Al-water reaction. Flow of thermal energy from the reaction was measured with a flexible ultrafast response heat flux sensor purchased from Shop RdF. Heat flux measurements submerged in water were performed using a custom setup incorporating a glass flange from Chemglass (ID: 27 mm, OD: 32 mm). Experiments controlling the rate of water were performed on an Instron 5942 Series Universal Testing System with a 500-N load cell. A Nordic Power Profiler Kit II was used to record the current output generated by the reaction, with results displayed in the nRF Connect for Desktop software interface. All heat characterization measurements were performed at room temperature, and all conditions were repeated in triplicate.

### Heat Flux Data Processing

The heat flux sensor captured an electrical signal proportional to the total heat rate applied to the surface of the sensor. Since the Nordic Power Profiler Kit II gave us a current readout, we converted the values to voltage output using Ohm’s law, so that we could ultimately calculate the heat flux by dividing that output by the sensor sensitivity per eq 2.1. The specifications provided by the sensor manufacturer indicated the sensor should provide an output of 6.9/3.155 μVm^2^/W at room temperature.
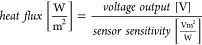


### Proof of Concept Actuation

To set the shape of nickel titanium alloy (Nitinol) wire, a custom fixture (Supplementary Figure 7C) inspired by previous literature was fabricated^64^ that consisted of an Al plate and a set of screws and hex nuts to tighten the wrapped-around wire and immobilize it for annealing at 400 °C for 20 min inside a high-temperature laboratory oven (LHT 6/30, Carbolite Gero). After heat treatment, the fixed Nitinol wire was quenched in room temperature water.

### Topology Optimization

To design a Nitinol hinge for our gastric device prototype, the Nitinol shape was obtained through the specific Kang and James topology optimization algorithm developed via Matlab simulation,^65^ based on the Lagoudas quadratic phenomenological constitutive model.^66^ The same material properties of Nitinol as tabled in the previous work^65^ were used for topology optimization, and readers of interest are referred to the literature for further details of the topology optimization algorithm. A brief description of the topology optimization algorithm used here can be found in Supplementary Discussion 3.

### Water Flow through Foam Characterization/Quantification

The impact of a water-limiting layer on the water flow rate was tested by using four foams with differing hydrophilicities. The foams were each cut or stacked into cylinders with a diameter of 3.2 cm and thickness of 1.25 or 2.5 cm and placed into a custom attachment that enabled the temporary attachment of the foams to the base of the glass flange. The two-part custom attachment was designed in SolidWorks (Student Edition 2020–2021) and printed in PLA by using a PRUSA i3MK3 3D printer. Two sets of four magnets (3 × 6 × 1.5 mm) were used to connect the top and bottom halves of the attachment, and the top half was hot glued directly to the base of the flange (Supplementary Figure 6D). To minimize leaks, a thin ring of hot glue was applied at the PLA-foam interface, as shown in Supplementary Figure 6E. The top of the flange was sealed using Parafilm M, 50 mL of water was added to the inverted flange, the foam containing attachment was magnetically attached, and a thin strip of Parafilm was wrapped around the magnetically attached interface to minimize water leakage. The flange setup was then flipped upright and clamped above a glass dish resting on a Mettler Toledo PJ600 balance. While filming the setup, the parafilm sealing the top of the flange was pierced to begin water flow through the foam. Kinovea (Version 0.9.5) was used to export screenshots showing the mass of water collected at a frequency of every 200, 500, or 1000 ms, depending on the hydrophilicity of the foam. All measurements for each foam type were acquired in triplicate.

### Prototype Fabrication

The Nitinol hinge was laser cut from 0.5 mm thick shape-memory Nitinol sheets (Black Cat Laboratories, Somerville, MA, USA). Al wire wrapped around the folded Nitinol structure was used to set the shape during the same heat treatment described above. The final prototype was assembled by attaching superelastic Nitinol arms to the shape-memory Nitinol hinge using Al wire and sealing the connection with superglue. The Nitinol arms were dipped in molten PCL at 80 °C to provide the coating. Dialysis tubing was wrapped around the center of the hinge three times and sealed with superglue on the edges. Lastly, heat-shrink tubing was slid around the ends of the arms and locally sealed with a heat gun (Wagner FURNO 300).

### *Ex Vivo* Testing

*Ex vivo* tests were performed to demonstrate the actuation of the single Nitinol component, both by dripping water on the component in air and when fully submerged in a beaker filled with room temperature water. Further tests involved the deployment of the prototype from a triple zero gelatin capsule (000) and recording its actuation performance. The latter was done by calipers to take measurements of the prototype dimensions when folded to assess the displacement, and the joint angles were analyzed in Adobe Illustrator from captured images.

### Animal Experiments

*In vivo* testing was conducted to evaluate the actuation viability of the prototype inside the stomach. All animal experiments were approved by the Committee on Animal Care at the Massachusetts Institute of Technology. *In vivo* swine experiments were performed in a terminal setting on Yorkshire pigs (80–100 kg) due to the similarity of their gastrointestinal tract (GIT) anatomy compared to that of humans. Pigs were anesthetized prior to endoscopy-assisted insertion of an overtube, as previously described,^64,67,68^ which was used to administer devices. An endoscope camera was used to record the administration, deployment, and actuation of the prototype.

## Supporting Information Available

Overview of pros and cons of conventional methods of heat generation; Al-grain boundaries penetration by stirred EGaIn; Surface temperature of reaction encapsulated in a semipermeable membrane (discussion and figure); Impact of pH and buffering on the activated Al-water reaction (discussion and figure); IR camera images for temperature peaks at different pH values; Sample setup for thermal measurements; Custom setup for water flow measurements of different foams; Topology optimization method; Nitinol manipulation; Topology optimization; Fabrication of Nitinol hinge; Battery specifications to calculate the total energy stored; Back-of-the-envelope comparison of how much power it takes to electrically heat Nitinol using resistive heating (PDF)
Simulated actuation of a Nitinol stent (MP4)
Simulated actuation of half of the optimized Nitinol hinge (MP4)
