# Supplementary material for: Activated Metals to Generate Heat for Biomedical Applications
Source: ACS Mater Lett. 2023 Aug 16;5(9):2508–17. doi: 10.1021/acsmaterialslett.3c00581 (PMC10481395; doi:10.1021/acsmaterialslett.3c00581)
Supplement: Supplementary file 1 — tz3c00581_si_001.pdf [file tz3c00581_si_001.pdf]

# Supporting Information

## Activated Metals to Generate Heat for Biomedical Applications

Eva Remlova[+1,3], Vivian Rachel Feig[+1,2], Ziliang Kang[1,4], Ashka Patel[1,5], Ian Ballinger[1], Anna Ginzburg[1,6], Johannes Kuosmanen[4], Niora Fabian[4,7], Keiko Ishida[1,2], Joshua Jenkins[1,4], Alison Hayward[1,4,7], Giovanni Traverso[1, 4]*

1. Division of Gastroenterology, Department of Medicine, Brigham and Women's Hospital, Harvard Medical School, Boston, MA, 02115, USA.

2. The David H. Koch Institute for Integrative Cancer Research, Massachusetts Institute of Technology, Cambridge, MA, 02139, USA.

3. Department of Health Sciences and Technology, Eidgenössische Technische Hochschule Zürich, Universitätstrasse 2, 8092 Zürich, Switzerland.

4. Department of Mechanical Engineering, Massachusetts Institute of Technology, Cambridge, MA, 02139, USA.

5. Department of Bioengineering, Northeastern University, Boston, MA, 02115, USA.

6. Department of Cell/Cellular and Molecular Biology, Northeastern University, Boston, MA, 02115, USA.

7. Division of Comparative Medicine, Massachusetts Institute of Technology, Cambridge, MA, 02139, USA.

Correspondence should be addressed to: ctraverso@bwh.harvard.edu, cgt20@mit.edu (G.T.)

[+] These authors contributed equally.

**Table of Contents** (in the order of occurrence in the Manuscript):

***Supplementary Table 1:*** *Overview of pros and cons of conventional methods of heat generation.* ............ 3

***Supplementary Figure 1.*** *Al-grain boundaries penetration by stirred EGaIn*........................................... 4

***Supplementary Discussion 1:*** *Surface temperature of reaction encapsulated in a semi-permeable membrane.* ........................................................................................................................ 4

***Supplementary Figure 2.*** *Surface temperature of reaction encapsulated in a semi-permeable membrane.*5

***Supplementary Discussion 2:*** *Impact of pH and buffering on the activated Al-water reaction.* ................ 5

***Supplementary Figure 3.*** *Impact of pH and buffering on the activated Al-water reaction.* ....................... 6

***Supplementary Figure 4.*** *IR camera images for temperature peaks at different pH values* ....................... 7

***Supplementary Figure 5.*** *Sample setup for thermal measurements.* .......................................................... 7

***Supplementary Figure 6.*** *Custom setup for water flow measurements of different foams.* ......................... 8

***Supplementary Discussion 3:*** *Topology optimization method.*.................................................................. 8

***Supplementary Video 1.*** *Simulated actuation of a Nitinol stent*.................................................................. 9

***Supplementary Figure 7.*** *Nitinol manipulation.* .......................................................................................... 9

***Supplementary Figure 8.*** *Topology optimization*........................................................................................ 10

***Supplementary Video 2.*** *Simulated actuation of half of the optimized Nitinol hinge.* .............................. 10

***Supplementary Figure 9.*** *Fabrication of Nitinol hinge.* ............................................................................ 10

***Supplementary Table 2:*** *Battery specifications to calculate the total energy stored.*............................... 11

***Supplementary Table 3:*** *Back-of-the-envelope comparison of how much power it takes to electrically heat Nitinol using resistive heating.*............................................................................................... 12

***References***........................................................................................................................ 12

**Supplementary Table 1:** Overview of pros and cons of conventional methods of heat generation.

| Method | Pros | Cons | References |
|---|---|---|---|
| Joule heating | high spatial resolution | high power requirement | [1–4] |
| light-activated (infrared, near-infrared) | high spatial resolution, rapid | challenging to introduce *in vivo* | [4–6] |
| alternating magnetic field | rapid | challenging *in vivo*, potentially unsafe | [4] |
| microwave field | rapid, uniform heating | challenging *in vivo*, potentially unsafe | [4] |
| ultrasonic heating | favorable range of energy penetration characteristics, ability to shape the energy deposition patterns | challenging spatio-temporal control, heat loss due to perfusion | [7–11] |
| solvent or solution-based reactions | low power requirement | low spatial resolution | [6] |

**Supplementary Figure 1.** Al-grain boundaries penetration by stirred EGaIn.

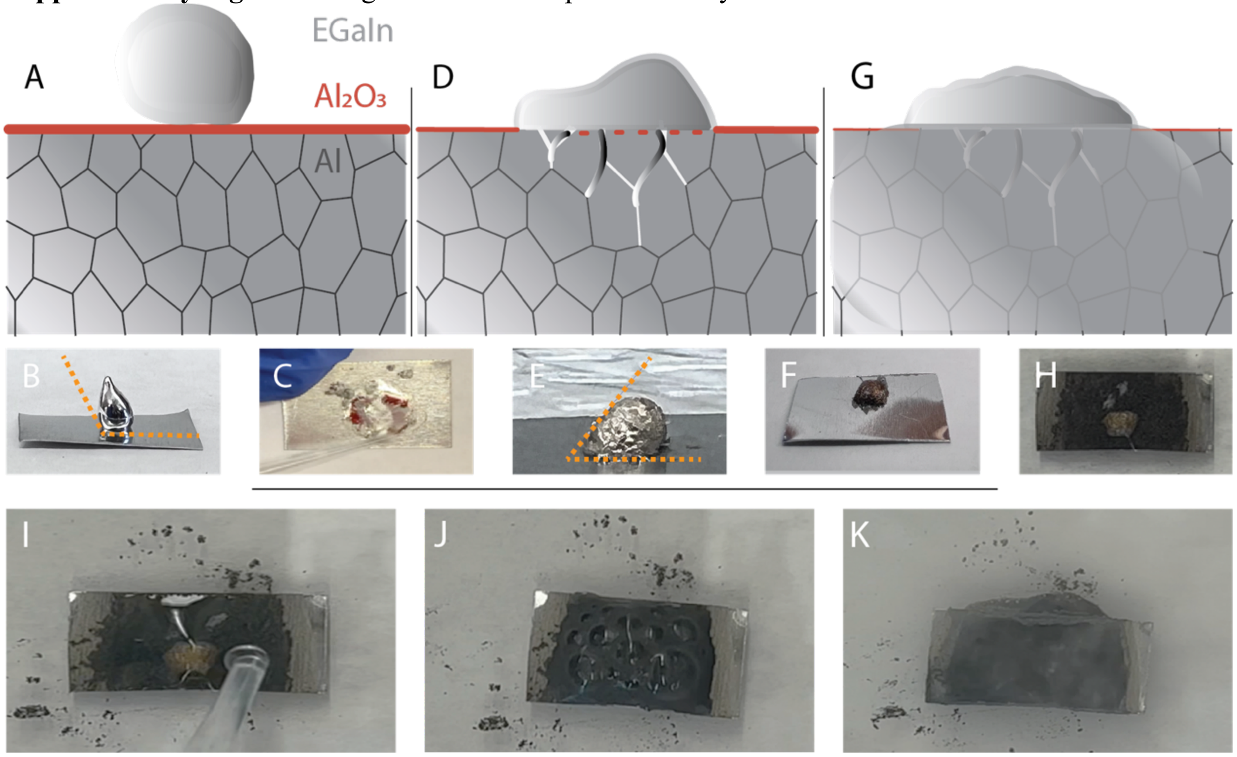


**Supplementary Figure 1: Al-grain boundaries penetration by stirred EGaIn:** (A) no wetting occurs, unstirred EGaIn cannot penetrate the Al surface oxide, and exhibits a high contact angle (B), (C) pipette tip can be used to mechanically agitate the EGaIn to promote its oxidation, and thus wettability (D), (E) reactive wetting: GB penetration is characterized by reduced contact angle and surface discoloration (F), (G) a quasi- uniform attack is evident as smooth-wide cavities around the initial contact area perimeter of the droplet [35], (H) Al-GB infused with EGaIn lead to the formation of EGaIn-Al amalgam and complete Al activation, and after adding water (I), activated Al can react with it (J) to generate heat (K)

**Supplementary Discussion 1:** Surface temperature of reaction encapsulated in a semi-permeable membrane.

Encapsulating the activated Al within a semipermeable membrane allows us to effectively leverage the exothermic Al-water reaction to actuate a thermally-responsive material to which the Al powder is thermally coupled. Meanwhile, the external surface of the membrane is able to maintain a safe temperature for surrounding tissues because of the efficient heat dissipation by the surrounding water, and by the further thermal insulation provided by the membrane. To illustrate this point, we used an IR camera to record the surface temperature of 15 mg of activated Al powder that was wrapped in dialysis tubing and submerged in water, repeating the experiment 3 times **(Supplementary Figure 2A, 2B)**. We compared this to 3 control runs, which consisted of dropping water onto 15 mg of activated Al in open air **(Supplementary Figure 2C, 2D)**. The peak temperatures recorded in the control cases were 84.1 °C, 96.7 °C, and 94.1 °C, whereas the peak temperatures recorded at the surface of the samples in the submerged and encapsulated case were 34.0 °C, 34.6 °C, and 31.7 °C. This illustrates that, despite the exothermic reaction occurring, the temperature at the surface remains safe to interface with tissue in the body's water-laden environment.

**Supplementary Figure 2.** Surface temperature of reaction encapsulated in a semi-permeable membrane.

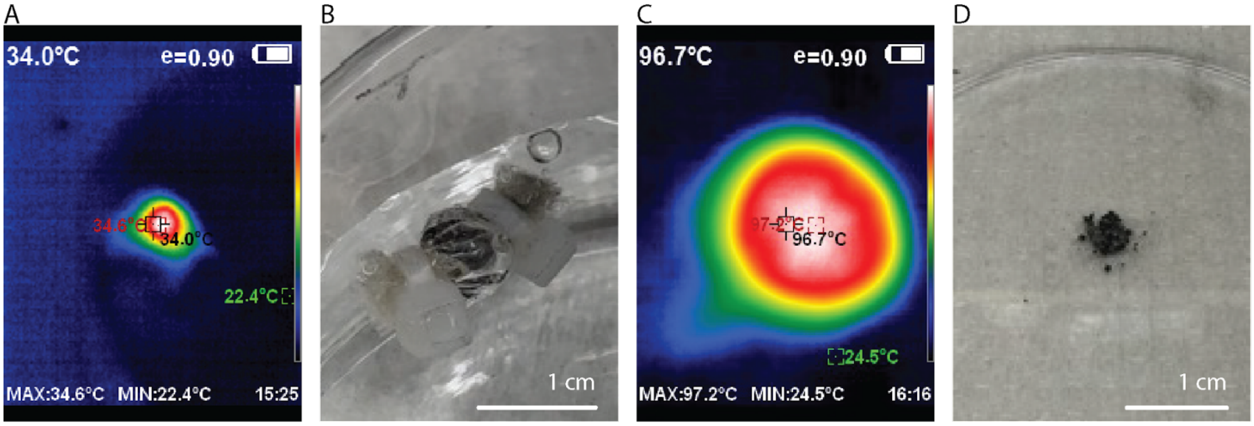


**Supplementary Figure 2: Surface temperature of reaction encapsulated in a semi-permeable membrane:** (A) temperature peak of activated aluminum packet after 8 minutes, (B) reacting aluminum powder encapsulated in two layers of dialysis membrane, (C) peak for the control samples (just 15 mg of powder reacted with 0.25 mL of water), (D) 15 mg of activated aluminum on a Petri dish

**Supplementary Discussion 2:** Impact of pH and buffering on the activated Al-water reaction.

We observed the reaction of activated aluminum in the presence of deionized water; simulated gastric fluid (SGF), which has a pH of 1.2 due to the presence of hydrochloric acid; simulated intestinal fluid (SIF), which is buffered to a pH of 6.8; and phosphate buffered saline (PBS) with a pH of 7.4 (**Supplementary Figure 3**). For each sample, 0.5 mL of the solution was added to a 1 $cm^2$ piece of activated aluminum in an open-air environment. We used a thermal camera to monitor the temperature of the system over time, recording the peak temperature and the time it took to reach the peak for each sample (**Supplementary Figure 4**).

The average peak temperature observed when samples were reacted with water was 92.0°C, and on average, it occurred 68.3 seconds after the start of the reaction. Similarly, when activated aluminum samples were reacted with SGF, the average peak temperature was 98.6°C, and the reaction reached its peak after an average of 90 seconds. These results indicate that a similar degree of thermal energy is released when activated aluminum interacts with highly acidic solutions, such as gastric fluid, compared to water.

When activated aluminum was reacted with buffered solutions, namely PBS and SIF, temperatures comparable to the Al-water reaction were achieved, however, the kinetics of the reaction differed significantly. In reactions occurring with SIF and PBS, the temperature increased at a slower rate than what was observed in non-buffered conditions, and two peaks were observed with the second peak driving the reaction to completion. The average temperatures of the first peaks were 60.8°C and 52.7°C for SIF and PBS, respectively, occurring 4.67 minutes and 11.83 minutes after the start of the reaction. After some time, the reactions displayed second peaks with average temperatures of 104.9°C and 102.3°C for SIF and PBS, respectively, at 67.0 minutes and 38.4 minutes. In essence, although the reaction follows different kinetics in buffered solutions, the generated heat is comparable to analogous non-buffered reactions.

We hypothesize that this difference in kinetics is observed as a result of the forced stability of the pH. When activated Al reacts with water, immediately prior to the sharp increase in temperature, there is a corresponding increase in pH of the reaction[15]. We experimentally verified this by sampling the pH using pH strips immediately after adding water, in the 40°C, and 70°C temperature range. The pH of the water

at the start of the reaction was 6, but it increased to 10 as the temperature began to ramp up and maintained this pH. From a theory perspective, OH serves as a catalyst in the aluminum and water reaction in accordance with the reaction scheme represented below[16]. Furthermore, the alkali concentration plays a crucial role in stimulating the chemical reactivity of Al, which is the first step required for the reaction to occur[16,17]. Essentially, higher concentrations of hydroxide promote the rapid generation of hydrogen and associated temperature increase.

$$2Al + 6\ H_2O + 2NaOH \rightarrow NaAl(OH)_4 + 3H_2 \qquad (1)$$

$$NaAl(OH)_4 \rightarrow NaOH + Al(OH)_3 \qquad (2)$$

$$2Al + 6\ H_2O \rightarrow 2\ Al(OH)_3 + 3H_2 \qquad (3)$$

**Supplementary Figure 3.** Impact of pH and buffering on the activated Al-water reaction.

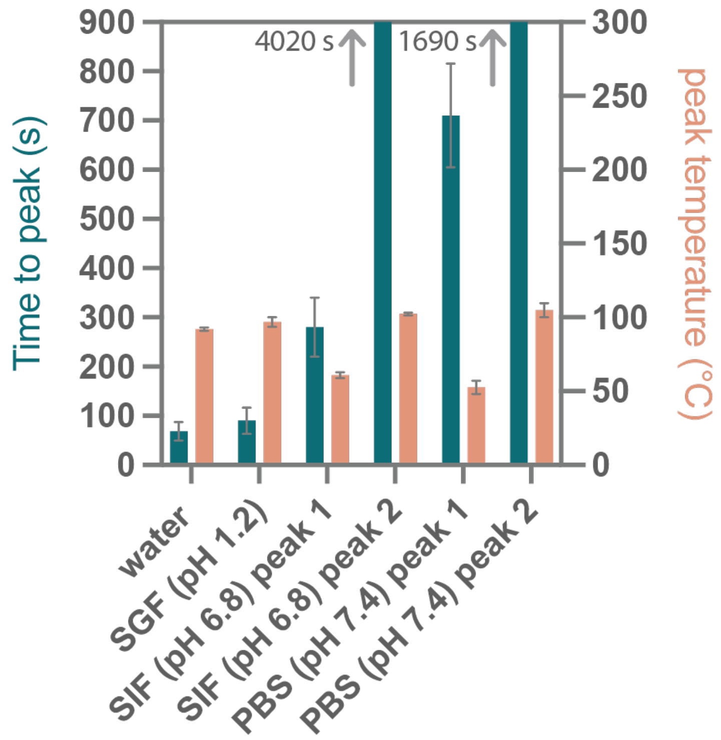


**Supplementary Figure 3: Impact of pH and buffering on the activated Al-water reaction:** in water (pH 7.1), simulated gastric fluid (SGF) and simulated intestinal fluid (SIF)

**Supplementary Figure 4.** IR camera images for temperature peaks at different pH values

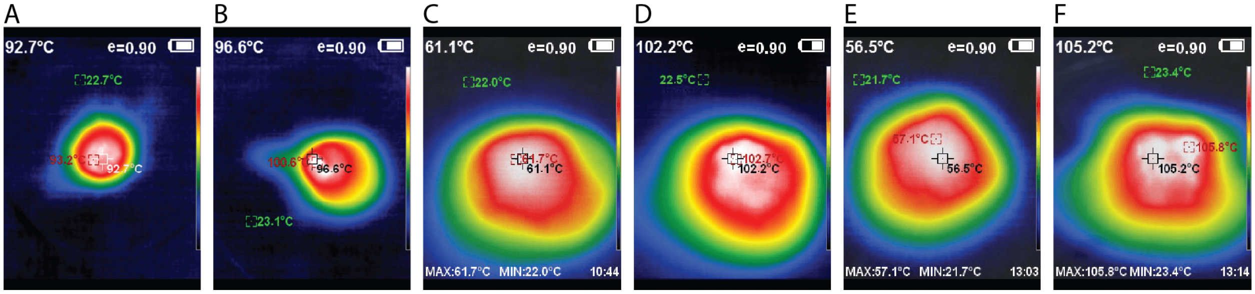


**Supplementary Figure 4: IR camera images:** (A) Water and (B) SGF at peak, SIF at (C) first peak and (D) second peak, PBS at (E) first peak and (F) second peak

**Supplementary Figure 5.** Sample setup for thermal measurements.

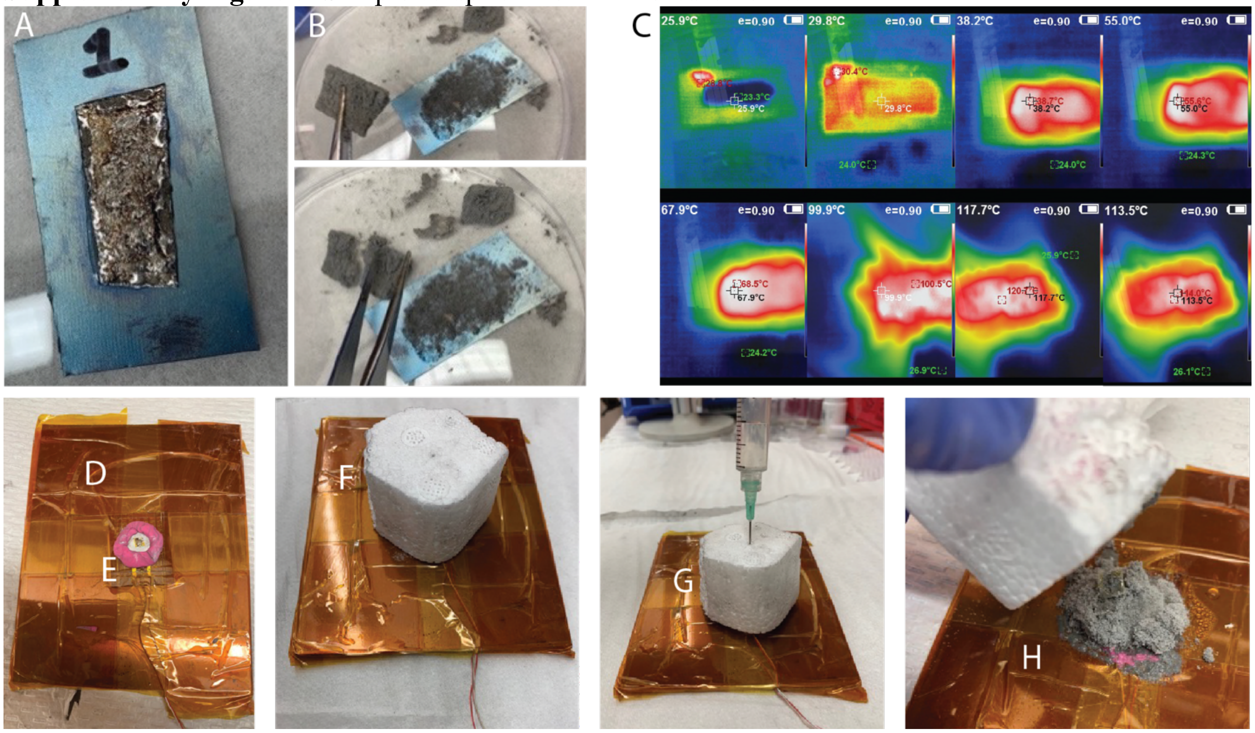


**Supplementary Figure 5: Sample setup for thermal measurements:**

**IR thermal measurements**: samples before (A) and after introducing water (B), (C) IR thermal camera imaging test sequence capturing the maximal temperature of the activated Al-water reaction

**HF measurements:** (D) heatsink insulated by Kapton tape to measure heat flux, with an exposed area for the sensor, (E) EGaIn- activated sample adhered to the sensor by thermal grease and mounted in place using thermal clay, (F) Styrofoam thermal insulation, (G) controlled water intro- duction drop by drop with a syringe, (H) at the end of reaction

**Supplementary Figure 6.** Custom setup for water flow measurements of different foams.

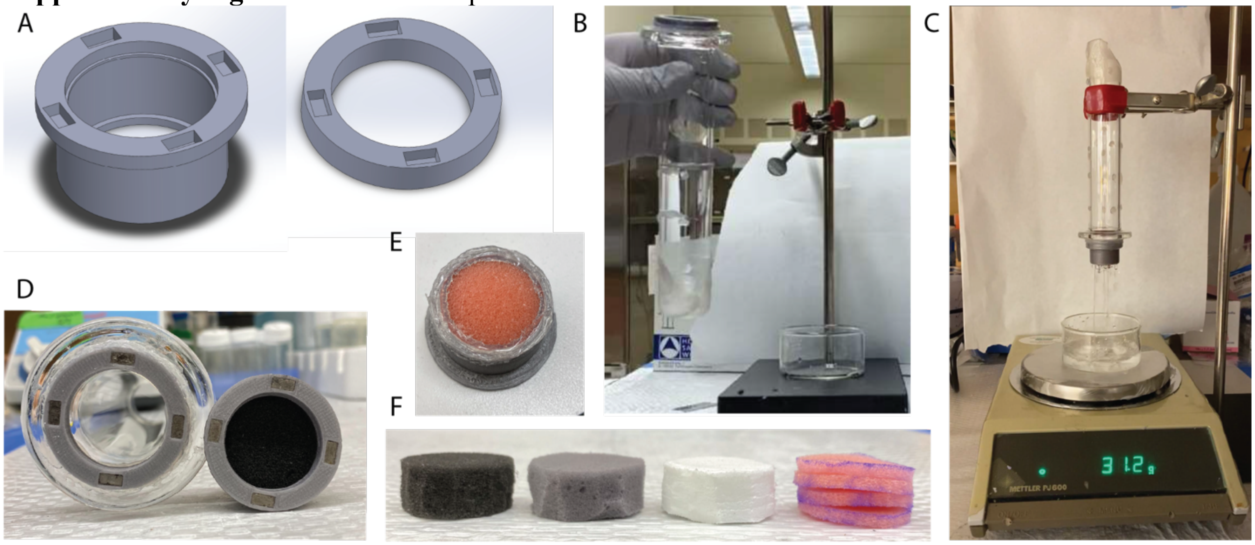


**Supplementary Figure 6: Custom setup for water flow measurements of different foams:** (A) Screenshots of the SolidWorks designs for the foam containing attachment, (B) The top of the column is parafilmed and water is added before the attachment containing the foam is clipped into place, (C) Experimental setup featuring the column with the foam contained within the PLA attachment, (D) Side-view of foam containing attachment in place with installed magnets to hold the halves together, (E) Foam encased in PLA attachment with a thin layer of hot glue overlaying the interface between the two materials, (F) Different foams tested, consistent in thickness and adjusted to fit inside the custom attachment

**Supplementary Discussion 3:** Topology optimization method.

For a detailed explanation of how topology optimization works for Nitinol, we refer the reader to reference [65] in the main text, as mentioned in the Methods section of our manuscript. Briefly, topology optimization allows one to optimally distribute materials within a design domain, based on the simulation of the response of a finite element mesh, which incorporates the thermomechanical properties of the material and is subject to various defined objectives and constraints. In this way, it is an inverse design method that allows one to generate a geometry or configuration for a material, absent any *a priori* shape or design.

The aim of the topology optimization approach we used was to maximize the displacement ($d_A$) of Nitinol in response to the heat flux, subject to several constraints: First, a volume constraint was applied to design the component to comprise less than 30% Nitinol; this is a common constraint in topology optimization in order to reduce manufacturing costs by reducing the amount of material used. Second, the topology optimization approach uses a finite element mesh to describe the design domain. Each element of the mesh is expected to be filled with a solid (Nitinol) material or void material. The compliance displacement constraint ($d_0$) is a common constraint used to avoid a conflicting presence of both solid and void materials within a single element, which will lead to an unphysical solution, and the choice of the upper bound of $d_0$ <= $2\text{x}10^{\wedge-3}$ mm was obtained through numerical trials by increasing the value until each element of the mesh is either only solid or only void material.

The thermomechanical properties of Nitinol were simulated using the Lagoudas model[12] relating $\sigma$ (the stress tensor of the design domain) to strain ($\varepsilon$) via $\varkappa$, the constitutive tensor described by the thermal dynamic transformation behaviors of Nitinol :

$$\sigma = \varkappa : \varepsilon$$

where

$$\varepsilon = \varepsilon^{e}(\sigma) + \varepsilon^{th}(T) + \varepsilon^{t}(\sigma, T, \xi)$$

The total strain is dependent on $\varepsilon^{e}$, $\varepsilon^{th}$ and $\varepsilon^{t}$ , which are the elastic strain tensor, pure thermal expansion tensor, and inelastic (transformation) strain tensor, respectively. Accordingly, the elastic strain $\varepsilon^{e}$ is affected by the pre-loading stress condition σ; the pure thermal strain $\varepsilon^{th}$ is affected by the temperature distribution on the Nitinol hinge, which is a function of the external heat flux and the thermal conductivity of Nitinol; the inelastic (transformation) strain $\varepsilon^{t}$ is affected by the pre-loading stress condition σ, the temperature $T$ and the volume fraction ratio of martensite lattice $\xi$ determined by thermal and mechanical energy that the lattice of Nitinol absorbed. Further detailed information of the phenomenological model was previously described in greater detail[13].

The result of the topology optimization yielded the structure shown in Supplementary Fig 5B. Mirroring this optimized structure yields a hinge design (Supplementary Fig 5C, Supplementary Video 2), which we incorporated as the central building block into more complex devices, including the gastric resident device that we demonstrated experimentally in Figure 3, and the thermally-responsive Nitinol stent that we computationally demonstrated in Supplementary Video 1.

**Supplementary Video 1.** Simulated actuation of a Nitinol stent.
Simulation of the thermally actuated collapse of a spherical stent in response to a temperature increase from the martensite phase to austenite phase of the studied Nitinol. The simulation was completed by implementing the Lagoudas model in COMSOL, considering a predefined temperature increase from 10°C to 60°C.

**Supplementary Figure 7.** Nitinol manipulation.

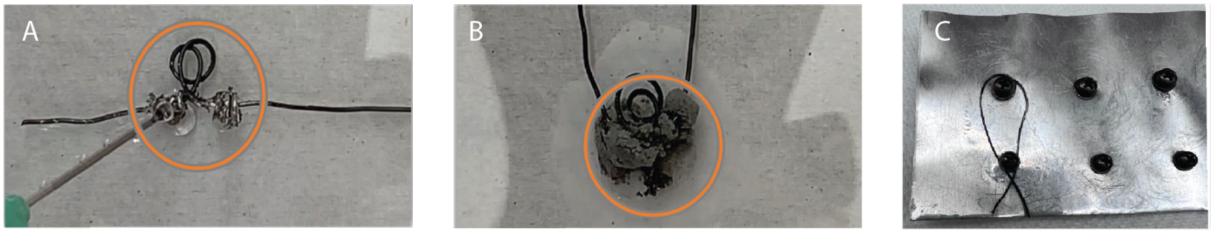


**Supplementary Figure 7: Nitinol manipulation:** (A) Triggering actuation: pulled open arms of the Nitinol spring were wrapped with activated Al wire that had been previously smeared with EGaIn; the pre-programmed shape of the Nitinol spring is to be recovered upon adding a few drops of water; (B) Proof of concept actuation; (C) Nitinol wire was set into the shape of a torsion spring using a custom fixture and annealed as such

**Supplementary Figure 8.** Topology optimization.

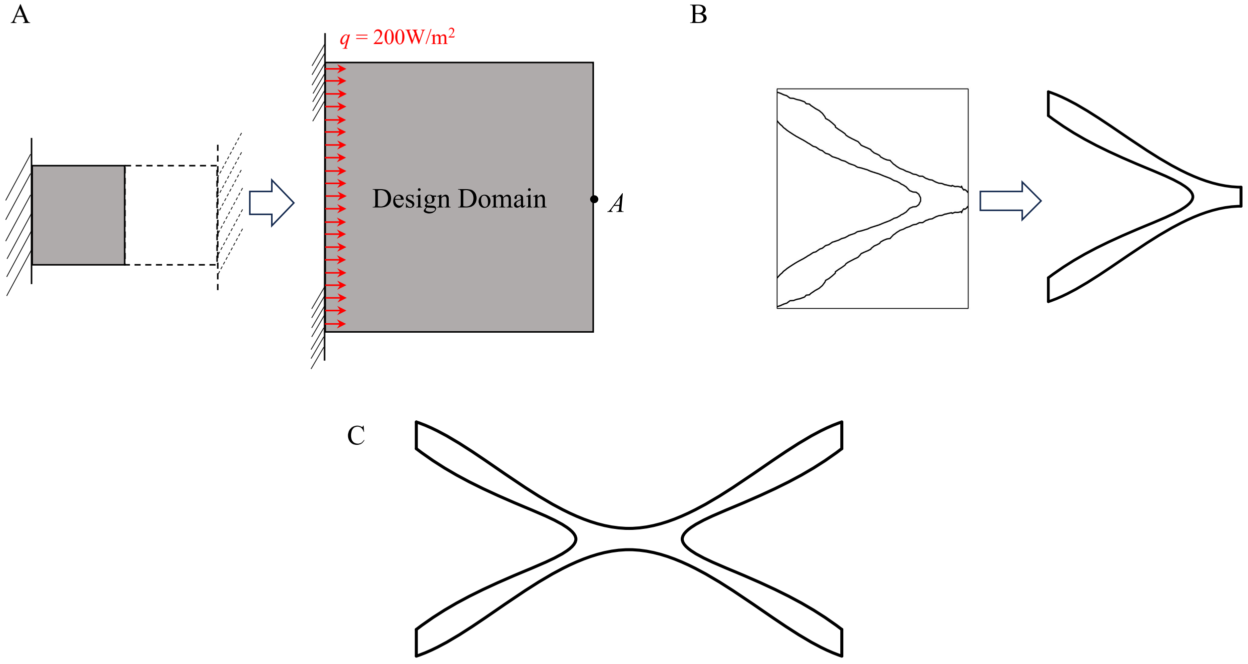


**Supplementary Figure 8: Topology optimization:** (A) Determine the design domain from a symmetric design problem, (B) Topology optimization result (left), smoothed geometry in Solidworks for laser cutting to avoid sharps in the *in-vivo* test (right), (C) Proposed Nitinol hinge shape

**Supplementary Video 2.** Simulated actuation of half of the optimized Nitinol hinge.
Simulation of the thermally actuated folding of half of the optimized hinge design in response to a temperature increase from the martensite phase to austenite phase of the studied Nitinol. The simulation was completed by implementing the Lagoudas model in COMSOL, with a predefined temperature increase from 10°C to 60°C.

**Supplementary Figure 9.** Fabrication of Nitinol hinge.

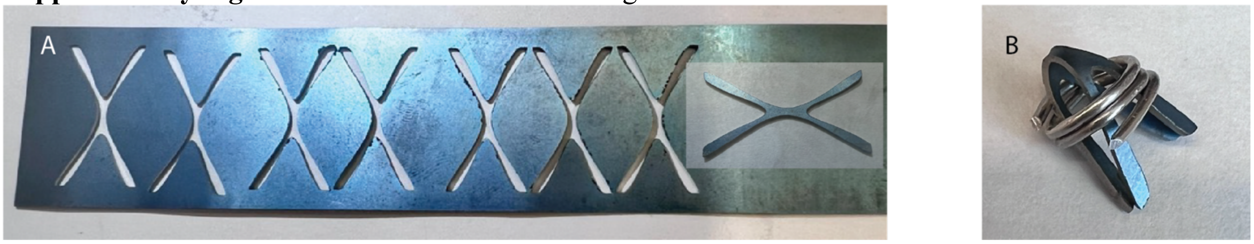


**Supplementary Figure 9: Fabrication of Nitinol hinge:** (A) Laser-cut hinge shape from a Nitinol sheet, (B) Custom fixture for annealing

**Supplementary Table 2:** Battery specifications to calculate the total energy stored.

| **Renata size 373 0% HG battery** *(manufactured by Renata, can be purchased from Mouser Electronics* [14] | **Specifications** |
|---|---|
| Voltage output | 1.55 V |
| Capacity | 29 mAh |
| Weight | 0.5 g |
| Width | 9.5 mm |
| Height | 1.65 mm |
| **000 capsule (26.14 mm x 9.91 mm)** | **Calculations** |
| we can fit: (capsule length/battery width) batteries | (26.14 mm/1.65 mm) ~ 15 batteries |
| *power capacity indicates how much energy is stored in a battery, often expressed in Watt-hours, and can be calculated as follows:* *Voltage * Amps * hours = [Wh] and since 1hour=3600 seconds* | |
| We can calculate stored energy in one battery as: voltage output * capacity * 3600: | 1.55 V * 29 mAh * 3600 = 161.8 Ws (equivalent to 161.8 J) |
| Total energy stored in the battery pack: | 15 * 161.8 Ws ~ 2.4 kWs |
| number of batteries * stored energy: | (equivalent to 2.4 kJ) |

**Supplementary Table 3:** Back-of-the-envelope comparison of how much power it takes to electrically heat Nitinol using resistive heating.

| Our Nitinol design | Measurements and Calculations |
|---|---|
| Weight | 0.127 g |
| Width at the center of actuation | 1.25 mm<br>(1 mm center, 1.5 mm edges, averaged: 1.25 mm) |
| Height | 0.5 mm |
| Cross-sectional area at the center of the hinge | 0.625 $mm^2$ (1.25 * 0.5mm) |
| Length | 5mm |
| Transformation temperature difference | from ambient (37°C) to transition temperature (45°C)<br>$\Delta t$=8°C |
| Latent heat ($H_L$) | 0.127 g * 20 J/g |
| Specific heat ($H_S$) | 0.127 g * 0.01 J/g * $\Delta t$ |
| **Total heat energy ($H_L + H_S$)** | ~2.55 J |
| Power required to cycle actuation in one second? (P=E * t) | 2.55 W |
| **Power amplification example** | |
| In comparison: *a gastric resident device that only needed 0.8 mW of power to corrode a membrane to release a drug* [2] | |
| **What would be the power amplification for our system if we could achieve the same actuation just by dissolving a membrane to let in water?** | 2.55 W/0.8 mW ~ 3190x amplification |

**References:**


(1) Wu, H.; Gao, W.; Yin, Z. Materials, Devices and Systems of Soft Bioelectronics for Precision Therapy. *Advanced Healthcare Materials* **2017**, *6* (10), 1700017. https://doi.org/10.1002/adhm.201700017.

(2) Kong, Y. L.; Zou, X.; McCandler, C. A.; Kirtane, A. R.; Ning, S.; Zhou, J.; Abid, A.; Jafari, M.; Rogner, J.; Minahan, D.; Collins, J. E.; McDonnell, S.; Cleveland, C.; Bensel, T.; Tamang, S.; Arrick, G.; Gimbel, A.; Hua, T.; Ghosh, U.; Soares, V.; Wang, N.; Wahane, A.; Hayward, A.; Zhang, S.; Smith, B. R.; Langer, R.; Traverso, G. 3D-Printed Gastric Resident Electronics. *Adv. Mater. Technol.* **2019**, *4* (3), 1800490. https://doi.org/10.1002/admt.201800490.

(3) Kalidasan, V.; Yang, X.; Xiong, Z.; Li, R. R.; Yao, H.; Godaba, H.; Obuobi, S.; Singh, P.; Guan, X.; Tian, X.; Kurt, S. A.; Li, Z.; Mukherjee, D.; Rajarethinam, R.; Chong, C. S.; Wang, J.-W.; Ee, P. L. R.; Loke, W.; Tee, B. C. K.; Ouyang, J.; Charles, C. J.; Ho, J. S. Wirelessly Operated Bioelectronic

Sutures for the Monitoring of Deep Surgical Wounds. *Nat Biomed Eng* **2021**, *5* (10), 1217–1227. https://doi.org/10.1038/s41551-021-00802-0.
(4) Wang, W.; Liu, Y.; Leng, J. Recent Developments in Shape Memory Polymer Nanocomposites: Actuation Methods and Mechanisms. *Coordination Chemistry Reviews* **2016**, *C* (320–321), 38–52. https://doi.org/10.1016/j.ccr.2016.03.007.
(5) Zhang, Y.; Wang, Z.; Yang, Y.; Chen, Q.; Qian, X.; Wu, Y.; Liang, H.; Xu, Y.; Wei, Y.; Ji, Y. Seamless Multimaterial 3D Liquid-Crystalline Elastomer Actuators for next-Generation Entirely Soft Robots. *Sci. Adv.* **2020**, *6* (9). https://doi.org/10.1126/sciadv.aay8606.
(6) Hines, L.; Petersen, K.; Lum, G. Z.; Sitti, M. Soft Actuators for Small-Scale Robotics. *Advanced Materials* **2017**, *29* (13), 1603483. https://doi.org/10.1002/adma.201603483.
(7) Kok, H. P.; Cressman, E. N. K.; Ceelen, W.; Brace, C. L.; Ivkov, R.; Grüll, H.; ter Haar, G.; Wust, P.; Crezee, J. Heating Technology for Malignant Tumors: A Review. *International Journal of Hyperthermia* **2020**, *37* (1), 711–741. https://doi.org/10.1080/02656736.2020.1779357.
(8) Ordeig, O.; Chin, S. Y.; Kim, S.; Chitnis, P. V.; Sia, S. K. An Implantable Compound-Releasing Capsule Triggered on Demand by Ultrasound. *Sci Rep* **2016**, *6* (1), 22803. https://doi.org/10.1038/srep22803.
(9) Miller, D.; Smith, N.; Bailey, M.; Czarnota, G.; Hynynen, K.; Makin, I. Overview of Therapeutic Ultrasound Applications and Safety Considerations. *J Ultrasound Med* **2012**, *31* (4), 623–634.
(10) Shin Low, S.; Nong Lim, C.; Yew, M.; Siong Chai, W.; Low, L. E.; Manickam, S.; Ti Tey, B.; Show, P. L. Recent Ultrasound Advancements for the Manipulation of Nanobiomaterials and Nanoformulations for Drug Delivery. *Ultrasonics Sonochemistry* **2021**, *80*, 105805. https://doi.org/10.1016/j.ultsonch.2021.105805.
(11) Diederich, C. J.; Hynynen, K. Ultrasound Technology for Hyperthermia. *Ultrasound in Medicine & Biology* **1999**, *25* (6), 871–887. https://doi.org/10.1016/S0301-5629(99)00048-4.
(12) Lagoudas, D. C. *Shape Memory Alloys: Modeling and Engineering Applications*; Springer US: Boston, MA, 2008. https://doi.org/10.1007/978-0-387-47685-8.
(13) Kang, Z.; James, K. A. Multiphysics Design of Programmable Shape-Memory Alloy-Based Smart Structures via Topology Optimization. *Struct Multidisc Optim* **2021**, *65* (1), 24. https://doi.org/10.1007/s00158-021-03101-z.
(14) *373.MP O% HG Renata | Mouser*. Mouser Electronics. https://www.mouser.com/ProductDetail/614-373-0HG (accessed 2022-06-20).
(15) Skrovan, J.; Alfantazi, A.; Troczynski, T. Enhancing Aluminum Corrosion in Water. *J Appl Electrochem* **2009**, *39* (10), 1695–1702. https://doi.org/10.1007/s10800-009-9862-x.
(16) Xu, S.; Zhao, X.; Liu, J. Liquid Metal Activated Aluminum-Water Reaction for Direct Hydrogen Generation at Room Temperature. *Renewable and Sustainable Energy Reviews* **2018**, *92*, 17–37. https://doi.org/10.1016/j.rser.2018.04.052.
(17) Liu, H.; Yang, F.; Yang, B.; Zhang, Q.; Chai, Y.; Wang, N. Rapid Hydrogen Generation through Aluminum-Water Reaction in Alkali Solution. *Catalysis Today* **2018**, *318*, 52–58. https://doi.org/10.1016/j.cattod.2018.03.030.
